# Portuguese Monofloral Honeys: Molecular Insights and Biochemical Characterization

**DOI:** 10.3390/molecules30081808

**Published:** 2025-04-17

**Authors:** Mariana Silva, Miguel Maia, Márcia Carvalho, Ana Novo Barros

**Affiliations:** 1 Centre for Research and Technology of Agro-Environment and Biological Sciences (CITAB), Institute for Innovation, Capacity Building and Sustainability of Agri-Food Production (Inov4Agro), University of Trás-os-Montes and Alto Douro (UTAD), 5000-801 Vila Real, Portugal; 2 APISMAIA, Produtos & Serviços, 4490-463 Póvoa de Varzim, Portugal; info@apismaia.com

**Keywords:** honey, geographical and botanical origin, phenolic compounds, antioxidant capacity, molecular marker

## Abstract

Honey is a multifaceted substance whose composition is intricately affected by various biotic and abiotic elements generated in the bee colony’s surroundings, including botanical and geographical origins, climatic conditions, soil characteristics, and beekeeping techniques. Monofloral honeys are identified by pollen analysis and are derived from the nectar of a predominant plant species, exhibiting rich sensory and nutritional profiles, making them food matrices with unique characteristics and excellent qualities. To explore the monofloral honey potential harvested in different regions of Portugal, a comprehensive study was conducted including the determination of phenolic composition and the assessment of biological activities. In addition to this evaluation, the inter simple sequence repeat (ISSR) was used to help differentiate honeys by botanical origin. The phenolic content and the antioxidant capacity were evaluated by spectrophotometric methods, observing, in general, differences between monofloral honeys. The honey from *Citrus sinensis* (Silves) exhibited the lowest phenolic content, including total phenols, *ortho*-diphenols, and flavonoids, whereas honeydew (Vinhais) showed the highest values. Regarding the antioxidant capacity, honey from *Lavandula stoechas* (Almodôvar) presented the lowest values, while honeydew (Vinhais) displayed the highest values for both DPPH and FRAP assays. In relation to the ABTS assay, the honey from *Metrosideros excelsa* (Aveiro) exhibited the lowest values, whereas the honey from *Eucalyptus* spp. (Arouca) showed the highest. The ISSR marker analysis allows the distribution of the samples based on the honey’s botanical origin, suggesting its potential role in honey authentication.

## 1. Introduction

Honey is a natural sweet substance produced by *Apis mellifera* bees that is composed of approximately 200 different components. It is a nutritional sweetener composed mainly of monosaccharides such as fructose and glucose, with the water content representing approximately 18% of its composition [1,2,3]. In small quantities, honey also contains additional substances that increase its quality and health benefits, including organic acids, minerals, vitamins, enzymes, proteins, amino acids, volatile compounds, and phenolic compounds [4,5].

Most of the biological effects of honey come from its secondary metabolites, specifically the phenolic compounds present in its composition [6,7]. The phenolic compounds are responsible for several health benefits including antioxidant, antimicrobial, anticarcinogenic, anti-inflammatory, antiatherogenic, and antidiabetic capacities. These compounds significantly influence the appearance and function of honey, and their characterization is important for studying the botanical and geographic origins of honey [8]. Polyphenols in honey are derived from the plants from which nectar is collected and through contact with propolis within the hive. The primary phytochemicals identified in honey are phenolic acids, flavonoids, and their derivatives [9]. These compounds have been identified as the primary contributors to the antioxidant capacity of honey, which is primarily linked to their ability to prevent and/or inhibit the reactive oxygen species (ROS) that cause oxidative stress [10]. Honey’s antioxidant activity varies greatly depending on its floral source. The main factor influencing honey’s antioxidant activity is its botanical origin [11].

Honey composition is intrinsically affected by various biotic and abiotic factors, including its botanical and geographical origin, climatic conditions, beekeeping practices, and the duration and conditions of storage [12]. Based on its botanical origin, honey can be called multifloral when it is produced from the nectar of several species [4,13] or monofloral when it contains around 45% pollen from one predominant species [14]. Portuguese monofloral honeys have a rich diversity attributed to the variety of the country’s melliferous flora. The three most representative monofloral honeys in Portugal are *Lavandula stoechas*, *Erica umbellata*, and *Castanea sativa*. However, other prominent monofloral honeys including those from *Rosmarinus officinalis*, *Arbutus unedo*, *Echium plantagineum*, *Mentha pulegium*, *Citrus sinensis*, *Carlina racemosa*, *Eucalyptus* spp., and *Helianthus annuus* are emerging [15].

In recent decades, several efforts have been developed to characterize honeys concerning botanical origin and to help with their authenticity. Recently, the scientific community started to explore DNA as a source of knowledge and an alternative to identify the botanical origins of honey [16,17,18]. The DNA present in honey is derived from different sources such as plants, virus, bacteria, microorganisms, and honey bees, resulting from the honeybees’ contact during foraging activities for nectar collection and environmental exploration [19]. The information required to determine the botanical and geographic origin can be found in the DNA [20]. Several methodologies are available to determine genetic diversity, namely morphological and molecular (biochemical and DNA-based) markers. Molecular markers are highly helpful for plant identification and the establishment of the relations between them, with the advantage of allowing direct comparison of genetic material, independent of environmental influences [20,21]. In the honey bee, several molecular marker techniques are still applied [22]. The inter simple sequence repeat (ISSR) marker is a simple, easy, and quick method that allows the identification of multiple loci without requiring knowledge of the genome sequence [23]. ISSRs are dominant markers dispersed throughout the plant genome, making them very useful in studies of genetic diversity [24,25]. To a first approach for Portuguese honey samples differentiation, the ISSR marker will provide a substantial amount of data at a reasonable cost [25] that allows us to explore the honey’s authenticity.

The main aim of this study was to explore the potential of monofloral honeys harvested in different regions of Portugal. This exploitation was assessed through chemical composition, particularly in terms of secondary metabolites. In addition to this evaluation, it became essential to identify and discriminate botanical varieties to help to differentiate honeys using molecular markers, namely the ISSR marker.

## 2. Results and Discussion

### 2.1. Phytochemical and Antioxidant Capacity Determination

The results for the total phenols, *ortho*-diphenols, and flavonoid contents in the honey samples are presented in Table 1.

Data analysis revealed significant differences (*p* < 0.001) in the levels of total phenols, *ortho*-diphenols, and flavonoids (Table 1). The mean values in the total phenolic content ranged from 20.13 ± 0.37 to 126.00 ± 0.29 mg GAE/100 g of sample (for samples Cit1 and Mel2, respectively). The total phenolic content was higher in honeydew samples, as well as in multifloral honeys and monofloral honeys from some floral species, such as *Castanea sativa*, *Erica umbellata*, and *Arbutus unedo*, collected in various geographical regions, with a notable predominance in the north and central regions.

Alves et al. [26] developed an analysis of monofloral honey samples collected from various Portuguese districts, some of which had the same botanical origin as those used in this study. Compared to the Alves et al. [26] study, the *Lavandula stoechas*, *Citrus sinensis*, and *Arbutus unedo* samples from the present study generally showed lower total phenolic concentrations. For example, the Lavandula honey samples (Lav3 and Lav6 from Faro) presented phenolic contents lower (28.88 ± 0.40 and 34.34 ± 0.21 mg GAE/100 g, respectively) than those reported by Alves et al. [26] (40.0 and 80.0 mg GAE/100 g). Since these samples have the same botanical and geographical origin, these differences could be attributed to variables such as climatic conditions and harvest year (2013 and 2023). The citrus and *Arbutus unedo* samples from the Algarve also showed a lower total phenolic content than those presented by Alves et al. [26]. In contrast, eucalyptus honey from Aveiro in the present study had a lower total phenolic content (36.61 ± 0.36 and 49.59 ± 0.32 mg GAE/100 g) than those from Braga in Alves’ study [26], which ranged between 60.0 and 80.0 mg GAE/100 g [26]. In the case of *Erica umbellata* honey, both studies reported high concentrations of total phenols, with the sample Eri from this study exhibiting a concentration of 95.72 ± 1.19 mg GAE/100 g, while the 2013 study showed values from approximately 60 to 140 mg GAE/100 g, although these comparison was performed with different geographical origins [26]. These results suggest that geographical origin may not influence the phenolic composition.

A similar pattern was reported by Fernandes et al. (2021) [27], who analyzed five Portuguese honeys from different botanical sources, including *C. sativa*, *Eucalyptus* spp., *C. sinensis*, *L. stoechas*, and *E. cinerea*. The total phenolic content in those samples was higher (103.9 ± 2 mg GAE/100 g, 111.6 ± 2 mg GAE/100 g, 34.8 ± 1 mg GAE/100 g, 55.4 ± 0.3 mg GAE/100 g, and 179.6 ± 14 mg GAE/100 g, respectively) than that observed in the samples of the same species analyzed in the present study. Ferreira et al. [28] analyzed three honey samples from different botanical origins, one of which was also analyzed in this work, namely the heather honey from Portela (72.78 ± 0.02 mg GAE/100 g). Comparing these results with the present work, the phenolic content of the heather honey was lower than that of the Eri sample (95.72 ± 1.19 mg GAE/100 g). A similar trend was observed in the study by Soares et al. [29], which analyzed 15 honey samples from distinct botanical origins and sources in Portugal’s northern and central regions. The current study analyzed some of these samples, namely honey from *E. umbellata*, *L. stoechas*, and *C. sativa* and showed higher total phenolic concentrations compared to the results reported by Soares et al. [29]. Conversely, Aazza et al. (2013) [30] analyzed 13 commercial honey from a supermarket in the Algarve, 5 of which shared the same botanical origin as those analyzed in this study, such as *A. unedo*, *C. sinensis*, *Eucalyptus* spp., and *L. stoechas*. For *A. unedo*, *C. sinensis*, and *Eucalyptus* spp. honey, and the total phenolic content in the commercial samples was higher than those analyzed in this study.

In a study led by Kaškonienė et al. [31], honey samples (including one sample of monofloral honey from *Tilia* spp.) were collected in Poland, and this Tilia honey exhibited a total phenolic content of 15.31 ± 5.5 mg GAE/100 g, which is lower compared to the Portuguese honeys (35.06 ± 2.11 and 31.48 ± 0.02 mg GAE/100 g) presented in this study. Can et al. [32] analyzed 13 varieties of monofloral honey from Turkey, including honey from *Trifolium* spp., revealing a total phenolic content of 25.53 ± 5.90 mg GAE/100 g, which is lower compared to the Portuguese honey samples (34.41 ± 0.43 and 45.32 ± 1.35 mg GAE/100 g). Vasić et al. [33] studied 64 honeydew honey samples collected from various regions of Croatia. The phenolic content of these honeydew honey species ranged from 57 ± 0.14 to 160 ± 0.20 mg GAE/100 g, these values being similar to the honeydew from Poland [31] and from this study (105.10 ± 0.96 and 126.00 ± 0.29 mg GAE/100 g; Table 1).

As shown in Table 1, the *ortho*-diphenols content values ranged from 19.73 ± 0.59 to 188.73 ± 1.07 mg GAE/100 g for the samples Cit1 and Mel2, respectively. Significant differences were observed in honey samples the same botanical origin, namely from the Lav5 and Lav2 samples, with the highest concentration obtained in the central region (Chamusca) and the lowest in the southern region (Almodôvar). In contrast, Lav5 did not differ significantly from Lav3, Lav4, and Lav6, mainly from the south of Portugal, except Lav3, which was from the northern region (Mirandela). Also, significant differences were identified between two samples of chestnut honey, Cast1 and Cast3, both from Viseu. The *ortho*-diphenol content in Cast1 was the lowest (61.18 ± 0.28 mg GAE/100 g), while Cast3 had the highest concentration (88.09 ± 0.9 mg GAE/100 g). These variations obtained in honeys with the same botanical origin can be explained by factors such as beekeeping practices, environmental conditions, or other variables, as already mentioned for total phenols.

The honey from *Erica umbellata* (Eri) showed a high *ortho*-diphenols concentration of 120.22 ± 6.39 mg GAE/100 g, which was significantly different (*p* < 0.001) from the remaining samples. On the other hand, the honey from *Metrosideros excelsa* (Met) showed a low concentration of 33.92 ± 0.08 mg GAE/100 g, with no significant differences compared to Til2 and Lav5, both of which also had low *ortho*-diphenols values.

Following the pattern obtained by total phenol content, the honeydew samples presented the highest value of *ortho*-diphenols compared to the other samples. The Mel2 sample has a higher *ortho*-diphenol concentration (188.73 ± 1.07 mg GAE/100 g) than the Mel1 sample (98.14 ± 0.88 mg GAE/100 g), with significant differences (*p* < 0.05). These differences may be plausibly explained by the distinct geographic origins of the samples, Vinhais and Almeida, respectively.

The multifloral honey samples, as already mentioned, are influenced by different pollen sources. The sample with the highest *ortho*-diphenol content was Mul5 (97.48 ± 2.02 mg GAE/100 g), from Mirandela (north), while the lowest content was Mul1(66.89 ± 1.10 mg GAE/100 g), from Freamunde (north). Both samples were collected in the north of Portugal, suggesting that the differences can be explained by their botanical composition.

The total flavonoid content analyzed in the twenty-nine honey samples ranged from 1.40 ± 0.13 to 19.43 ± 0.42 mg CE/100 g from the samples Cit1 and Mel2, respectively (Table 1). In general, significant differences were not observed (*p* < 0.05) between honey samples with the same botanical origin. This emphasizes the importance of botanical origin in determining the flavonoid content, regardless of geographical location.

The *C. sinensis* honey samples (Table 1), as well as the total phenols and *ortho*-diphenols content, have the lowest values. In contrast, the honeydew samples exhibited the highest values for total phenols, *ortho*-diphenols, and flavonoids. Two honeydew samples presented significant differences (*p* < 0.05), Mel1 contains 14.49 ± 0.24 mg CE/100 g of flavonoids, while Mel2 19.43 ± 0.42 mg CE/100 g has the highest sample values in the study. These samples have different geographic locations, Mel1 being from Almeida and Mel2 from Vinhais. The fact that both samples are sourced from different regions, which have different climatic conditions, soil composition, flora diversity, and other factors, can be related to the differences observed in flavonoid content.

Regarding the multifloral honey, the flavonoid content ranged from 10.45 ± 1.17 to 5.24 ± 0.28 mg CE/100 g from the samples Mul2 and Mul3, respectively. As shown in Table 1, Mul2 and Mul5 have similar values of flavonoid content, showing significant differences compared to the other multifloral honey samples.

The literature provides limited information on the content of flavonoids in Portuguese honey samples. However, Ferreira et al. [28] analyzed three honey samples, one of which had the same floral origin as the samples analyzed in this work, namely *Erica* spp. from Portela. In their analysis, the flavonoid concentration for the *Erica* spp. honey sample was 58.74 ± 0.05 mg CE/100 g, while in the present study, a concentration of 8.68 ± 0.37 mg CE/100 g was obtained for *E. umbellata* honey from Seia. These differences may be attributed to different variations, namely harvest year and regional climatic conditions.

The biosynthesis of phenolic compounds in plants varies significantly among species and is tightly regulated by genetic and environmental indications. For example, the phenylpropanoid pathway, which leads to the formation of flavonoids and phenolic acids, is more active in certain plant species depending on their defense mechanisms and stress responses [6,34].

Additionally, environmental factors such as sunlight exposure, temperature, soil composition, and water availability can modulate the expression of genes involved in secondary metabolism in plants, thus altering the phenolic profile of the nectar and consequently of the honey derived from it [10,34,35,36].

Seasonality and geographical location may also play key roles. For instance, honeys derived from plants growing in regions with higher UV radiation or drought stress may contain higher levels of specific antioxidants, as these compounds help the plant to cope with abiotic stress [37,38].

Although some differences in total phenolic content were observed among samples from different geographical regions, these variations cannot be solely attributed to geographical origin. The influence of geographical location is often intertwined with botanical origin, local environmental conditions, and beekeeping practices, making it difficult to isolate their individual effects. In this study, the botanical origin of honey samples emerged as a key factor influencing phenolic and flavonoid profiles, but climate, soil composition, and flora diversity are also known to modulate nectar composition and, consequently, the chemical characteristics of honey [39,40].

Notably, many of the sampled regions in Portugal share similar pedoclimatic conditions, which may explain the limited influence of geographical origin observed. All honey samples were harvested in the same production year (2022) and stored under controlled conditions (4 °C, protected from light), ensuring sample stability and data reproducibility. Although soil and climate parameters were not directly assessed in this study, we acknowledge their potential role and recommend future investigations with more controlled environmental and agronomic variables to further elucidate these influences.

### 2.2. Evaluation of Antioxidant Capacity

The presence of flavonoids and other phenolic compounds in honey is primarily responsible for its antioxidant properties. These properties are influenced by diverse factors, namely the botanical origin of the honey, environmental and seasonal variations, the packaging material, processing methods, and storage conditions. Such factors may explain the differences observed in the antioxidant capacity [5]. Additionally, the geographical origin and the year of harvest may have further influenced the observed variations in the results. The results for the antioxidant capacity for the twenty-nine honey samples, obtained using three methodologies, 2,2′-azino-bis (3-ethylbenzothiazoline-6-sulphonic acid (ABTS), 2,2-diphenyl-1-picrylhydrazyl reduction (DPPH), and Ferric Reducing Antioxidant Power (FRAP), are presented in Table 2. Data analysis revealed significant differences between samples in the three methodologies.

Significant differences (*p* < 0.05) were observed for the three antioxidant capacity parameters. For the ABTS assay, the mean values ranged from 0.038 ± 0.004 mmol T/100 g (Met) to 0.80 ± 0.03 mmol T/100 g (Euc2). The DPPH mean values ranged between 0.022 ± 0.002 mmol T/100 g (Lav5) and 0.47 ± 0.004 mmol T/100 g (Mel2), while the FRAP mean values varied from 0.046 ± 0.002 mmol T/100 g (Lav5 and Cit1) to 0.77 ± 0.03 mmol T/100 g (Mel2) (Table 2).

In the analysis of six honey samples from *L. stoechas* (Lav) compared to other honeys, the Lav samples generally demonstrated the lowest antioxidant capacity in both DPPH and FRAP assays, except the samples Lav2 and Lav6 which exhibited higher results for these two methodologies. However, no significant differences were observed between them in the FRAP assay. On the other hand, in the ABTS assay, the *L. stoechas* honey samples showed a higher capacity, with Lav1 and Lav2 displaying the highest results (0.38 ± 0.02 and 0.30 ± 0.02 mmol T/100 g, respectively). This result could indicate that honey may have additional components or factors influencing its antioxidant properties.

Regarding the five chestnut honey samples, the antioxidant capacity presented some variations, with samples exhibiting a higher antioxidant capacity in the FRAP assay, namely Cast3 and Cast4 (0.47 ± 0.01 and 0.38 ± 0.05 mmol T/100 g, respectively). In the ABTS assay, Cast5 showed the highest antioxidant capacity (0.53 ± 0.02 mmol T/100 g), significantly differing from the remaining samples. In the DPPH assay, Cast3 and Cast5 exhibited the highest antioxidant capacity (0.14 ± 0.004 and 0.18 ± 0.002 mmol T/100 g, respectively), while Cast1 and Cast2 exhibited lower antioxidant capacities and no significant differences in any assays.

The two honey samples from *Tilia* spp. (Til) showed the same tendency in antioxidant capacity for the ABTS and DPPH methodologies but exhibited differing results in the FRAP assay.

Regarding the two samples from *C. sinensis* (Cit), Cit2 demonstrated the highest antioxidant capacity across all three assays (0.12 ± 0.01, 0.12 ± 0.01, and 0.08 ± 0.00 mmol T/100 g, to ABTS, DPPH, and FRAP, respectively), with no significant differences. In contrast, Cit1 shows a lower antioxidant capacity in all three methodologies, corroborating the data obtained by the total phenols, *ortho*-diphenols, and flavonoids analyses.

In the two honey samples from *Trifolium* spp. (Tri), the DPPH and FRAP assays showed similar trends, with Tri1 exhibiting a higher antioxidant capacity (0.12 ± 0.01 and 0.13 ± 0.01 mmol T/100 g, respectively), with no significant differences. The sample Tri2, on the DPPH assay, shows the lowest antioxidant capacity (0.06 ± 0.00 mmol T/100 g), with no significant difference from Euc1 and Lav1. On the other hand, in the ABTS assay, Tri1 and Tri2 exhibited the same antioxidant capacity (0.19 ± 0.01 mmol T/100 g), making these results consistent with the phenolic composition of the samples.

Considering the *E. umbellata* honey sample, a slightly higher antioxidant activity was expected based on the phenolic content results shown in Table 1.

The honeydew sample presented the highest values of antioxidant capacity in the three assays, which was expected since it shows the highest values for total phenols, *ortho*-diphenols, and flavonoid content. However, in the ABTS assay, the sample Mel2 showed a lower antioxidant capacity (0.19 ± 0.01 mmol T/100 g) than expected. For other hand, the honey samples from *A. unedo* revealed a high antioxidant capacity in all three methodologies (Table 2), as expected with the phenolic content presented in Table 1.

The honey sample from *M. excelsa* exhibits a low antioxidant capacity across all three methodologies, where the results are aligned with the results obtained for phenolic content (Table 1).

Considering the multifloral honey samples, in the ABTS assay, Mul2 and Mul4 showed the highest antioxidant capacities (0.40 ± 0.01 and 0.38 ± 0.02 mmol T/100 g, respectively). Similarly, in the DPPH assay, Mul2 and Mul5 showed the highest antioxidant capacities (0.20 ± 0.00 and 0.20 ± 0.01 mmol T/100 g, respectively). On the other hand, in the FRAP assay, all samples had significant differences (*p* < 0.05), with Mul5 showing the highest antioxidant capacity (0.51 ± 0.01 mmol T/100 g). These variations may be attributed to several factors, particularly the geographical origin of the honey samples.

To facilitate the comparison and interpretation of the results comparative to other studies, the values obtained for ABTS and DPPH were converted into percentage inhibition (Table 2).

Alves et al. [26] analyzed the DPPH assay for 39 honey samples from nine Portuguese districts, and the results obtained for *L. stoechas* and *C. sinensis* honey samples were similar to those obtained in our study. In the study by Gonçalves et al. [41], commercial Portuguese honeys were analyzed, namely honey from *Citrus* spp., *Lavandula* spp., *Eucalyptus* spp., and *Erica* spp. The DPPH assay results were higher than those obtained in our study, with both heather honey samples showing a higher antioxidant capacity, reported as 32.7% by their group compared to 23.21% in our study. In both studies, honey from *Erica* spp. exhibited a higher ferric reduction power, followed by eucalyptus honey. The honey from *Erica* spp. demonstrated the highest DPPH scavenging activity and the highest reducing power according to FRAP values when comparing the two studies. These results are probably attributed to its high phenolic content. Escuredo et al. [42] analyzed honey samples from *C. sativa* (chestnut), *Eucalyptus* spp., *Erica* (heather), and honeydew honey, demonstrating higher DPPH assay values than those obtained in our study. Honeydew had the highest antioxidant capacity (65.4%), while the other honey presented lower values, namely chestnut honey (53.5%), eucalyptus honey (25%), and heather honey (40.9%). However, in our study the honey samples showed lower antioxidant capacities, ranging from 7.38% to 17.03% for chestnut honey, 5.63% to 14.56% for eucalyptus honey, and 23.21% for heather honey. A similar pattern was observed by the Perna et al. [43], where the analyzed chestnut honey samples exhibited a higher inhibition percentage in the DPPH assay (75.37%) compared to those presented in our study (7.38% to 17.03%). Perna et al. [43] also analyzed eucalyptus and citrus honey, with resulting antioxidant capacities of 73.04% and 55.06%, respectively. In contrast, the Portuguese samples showed lower antioxidant capacities, ranging from 5.63% to 14.56% for eucalyptus honey and 2.22% to 11.56% for citrus honey.

Brazilian eucalyptus honey samples presented a higher antioxidant capacity for the ABTS assay than those of the present study, ranging between 0.31 ± 0.01 and 0.70 ± 0.03 mmol T/100 g for Brazilian samples and from 0.24 ± 0.003 to 0.80 ± 0.03 mmol T/100 g for Portuguese samples. The same group analyzed the antioxidant capacity in the FRAP assay, with Brazilian eucalyptus honey samples showing higher results ranging from 0.21 ± 0.00 to 0.39 ± 0.01 mmol T/100 g when compared to the eucalyptus honey in the current study (ranging between 0.12 ± 0.00 and 0.15 ± 0.00 mmol T/100 g) [44].

Castiglioni et al. [45] analyzed 117 monofloral honey samples, including honey from *Citrus* spp., *Eucalyptus* spp., *A. unedo* (strawberry tree), chestnut, and honeydew honey. Comparing both studies, the ABTS results obtained by Castiglioni et al. [45] showed that *A. unedo* honey exhibited the highest antioxidant capacity, followed by honeydew honey, eucalyptus honey, chestnut honey, and citrus honey. Similarly, in our study, *A. unedo* honey showed higher values, as observed in the Italian study, followed by eucalyptus honey, honeydew honey, chestnut honey, and citrus honey. In the DPPH assay, *Arbutus unedo* honey demonstrated the highest antioxidant capacity, followed by honeydew, while in our study, the honeydew demonstrated the highest value, followed by the strawberry tree honey sample. Jerković et al. [46] studied the radical scavenging capacity of *Trifolium* spp. honey through a DPPH assay. They demonstrated an antioxidant capacity of 23.3%, which is higher than the values observed in the present study (7.32% and 10.59%). FRAP was also measured in the honey sample, exhibiting a FRAP value of 0.059 mmol T/100 g for a 10% honey solution, which is lower than the FRAP results obtained in the present study (0.083 ± 0.00 and 0.131 ± 0.01 mmol Trolox/100 g).

Dzugan et al. [47] studied the antioxidant capacity of *Tilia* spp. honey using both the DPPH and FRAP assays. Their study revealed a 40.53% of radical scavenging capacity by DPPH assay, while in our study were observed lower values (ranging from 7.87% to 13.47%). On the other hand, the FRAP assay results showed that the Portuguese samples exhibited higher antioxidant capacities, ranging from 0.15 ± 0.001 to 0.20 ± 0.02 mmol Trolox/100 g, compared to the Dzugan et al. [47] study, which showed a lower value of 0.11 ± 0.03 mmol T/100 g. Studies realized by Honey et al. (2018) [48] on Portuguese *Arbutus unedo* honey samples demonstrated a slightly higher DPPH scavenging capacity (ranging from 40.28% to 45.20%) compared to our samples (35.85%).

To date, no studies on the antioxidant capacity of *Metrosideros excelsa* honey have been published, making this the first report on its properties.

### 2.3. Discrimination of Honey Samples by ISSR Marker

The sample of *Metrosideros excelsa* was quantified and showed a certain concentration of DNA but was excluded from the analysis of the molecular markers due to the absence of amplification in all the primers tested. This situation may be explained by the presence of PCR inhibitors or some DNA degradation that occurred after the DNA elution step. In future studies and DNA honey extractions will be important to wash the DNA well and immediately store it at −20 °C.

**Figure 1:**
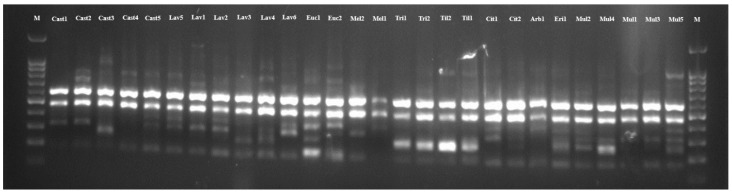
Agarose gel of primer UBC-840. Cast—*Castanea sativa*; Lav—*Lavandula stoechas*; Euc—*Eucalyptus* spp.; Mel—honeydew; Tri—*Trifolium* spp.; Til—*Tilia* spp.; Cit—*Citrus sinensis*; Arb—*Arbutus unedo*; Eri—*Erica umbellata*; Mul—multifloral; M—DNA marker GeneRuler 100 bp.

Honey botanical origin characterization using molecular markers is an important strategy to achieve honey authenticity. A simple methodology will supply reliable information about botanical origin, and the ISSR markers can be a first insight for this type of analysis due to their characteristics mentioned above. To explore this idea, a total of 25 ISSR primers were tested, and the 11 primers that were the most polymorphic and reproducible were selected as the most suitable for the genetic diversity analysis in twenty-eight honey samples (Figure 1).

These primers produced 118 markers among the genotypes, and the amplified products ranged from 200 bp to 2000 bp (Table 3). An average of 97.73% polymorphism was determined per primer and ranged from 75% (UBC-840) to 100% (the remaining primers). The number of polymorphic bands per primer varied between 9 (UBC-824, UBC-826, UBC-840, UBC-873, and UBC-891) and 14 (UBC-836), with an average of 10.45 bands per primer.

ISSR primers are dominant genetic markers widely distributed across the plant genome and are highly valued in genetic diversity studies. Primers with higher Rp values are usually more effective at identifying a greater number of genotypes and producing a higher number of polymorphic bands [24,49]. The primer UBC-888 was the most effective primer with a value of Rp of 9.71, and the Rp values ranged from 4.29 (UBC-857) to 9.71 (UBC-888), with an average of 6.94. The PIC of a molecular marker reflects its ability to identify polymorphisms between individuals in a population. A higher PIC value indicates a greater ability to reveal genetic variability and is therefore a key indicator of marker quality in genetic studies, whereas MI is used to assess the global utility of each marker system [24,50]. The PIC average in this study was 0.35, with the highest PIC at 0.43 (UBC-888) and the lowest at 0.27 (UBC-857), whereas MI ranged from 22.00 for UBC-840 to 42.72 for UBC-888 with an average of 34.33. The UBC-888 primer seems to be the most appropriate in this set of primers since it has the highest Rp, PIC, and MI values.

To the best of our knowledge, there are no studies on honey characterization through ISSR molecular markers; consequently, no data are available to discuss. However, a study of ISSRs was carried out on the different species used in this research. In the present study, the ISSR PIC value was 0.35, and the percentage of polymorphism was 97.73%, which are both higher than the values reported for *L. stoechas* (PIC = 0.25, %P = 49.0%) by Hmissi et al. [51]. In the case of *Castanea* spp., the PIC values and the percentage of polymorphism (0.36 and 97.14%, respectively) reported by Coutinho et al. [52] are similar; however, the Rp value (3.73) is lower than the value obtained in this study. Abdelhamid et al. [53] obtained lower MI values and percentages of polymorphism (5.57 and 79.2%, respectively) than the values in this study, while the PIC value (0.67) was higher. In *Eucalyptus* spp., the PIC value of 0.66 reported by Teixeira et al. [54] was higher than the average PIC value in this study (0.35). However, this value is from *E. microcorys* and *E. urophylla*, which may explain these differences comparative to this study. Similarly, in the case of *Trifolium* spp. as reported by Hmissi et al. [51], the PIC value (0.41) is similar to the PIC value found in this study (0.35).

Bands that consistently appear in all samples of one species but are absent in another can be regarded as potential species-specific markers. These markers can differentiate among the same species, and in the case of honey, it is a quick way of identifying pollen species [24]. Single bands are useful for identifying honeys, which means that if a single band appears in the samples from the same species, it is a band specific to this species. Seven unique bands were observed, three in a sample of *A. unedo*, one in *Eucalyptus* spp., one in multifloral honey, one in *Castanea sativa*, and one in *L. stoechas*. The highest number of unique bands was detected with UBC 857 and UBC 811 (two bands in each primer), while the primers UBC 873, UBC 824, and UBC 810 only presented a unique band.

**Figure 2:**
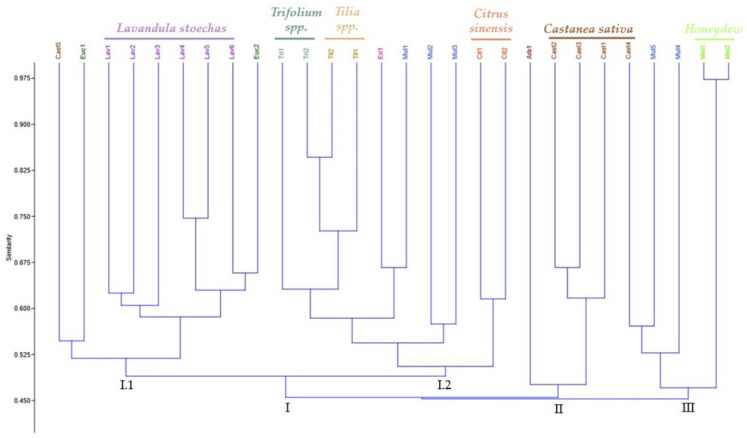
Dendogram estimating the genetic distance between the honey samples based on eleven ISSR markers.

Clustering patterns were obtained from the UPGMA cluster analysis of ISSR data (Figure 2), where the honey samples were grouped into three distinct clusters (I, II, and III). Generally, the honey samples were grouped based with their botanical origin, and the similarity coefficient varied between 0.450 and 0.97. The dendrogram showed that the honey samples of *L. stoechas*, *Trifolium* spp., *Tilia* spp., *C. sinensis*, *C. sativa*, and honeydew are grouped closely, except samples 4 and 5 of *Castanea sativa*. This indicates that honey samples could be differentiated based on their botanical origin, and the geographical origin did not interfere with the distribution.

The similarity values within cluster I ranged from 0.49 to 0.84 and could be further divided into two sub-clusters. In sub-cluster I-1, the *Lavandula stoechas* samples are closely grouped, while sub-cluster I-2 contains the samples of *Trifolium* spp., *Tilia* spp., and *Citrus sinensis*. In this case, Tri2 and Til2 are closer in genetic terms and are, therefore, similar. Similarly, the samples Eri1 and Mul1 are closely related. Given that multifloral honey is derived from various botanical sources, the Mul1 sample may contain a certain percentage of monofloral honey from *Erica umbellata* (Eri1), contributing to their similarity.

In cluster II, the coefficient of similarity varied between 0.490 and 0.675 and included four samples of monofloral honey, with the *Castanea sativa* samples grouped, except for the sample Cast5. The third group consists of five samples with similarity values between 0.490 and 0.980. The samples Cast4 and Mul5 show the same degree of similarity; however, the composition of the Mul5 is unknown suggesting that this sample contains a certain percentage of monofloral honey from *Castanea sativa*. The honeydew samples are the most distinct from the other samples; however, they are the most similar to each other. This distinction may be due to the different genetic material present in honeydew since it is derived from insects.

### 2.4. Integrative and Correlation Analysis of All Parameters

**Figure 3:**
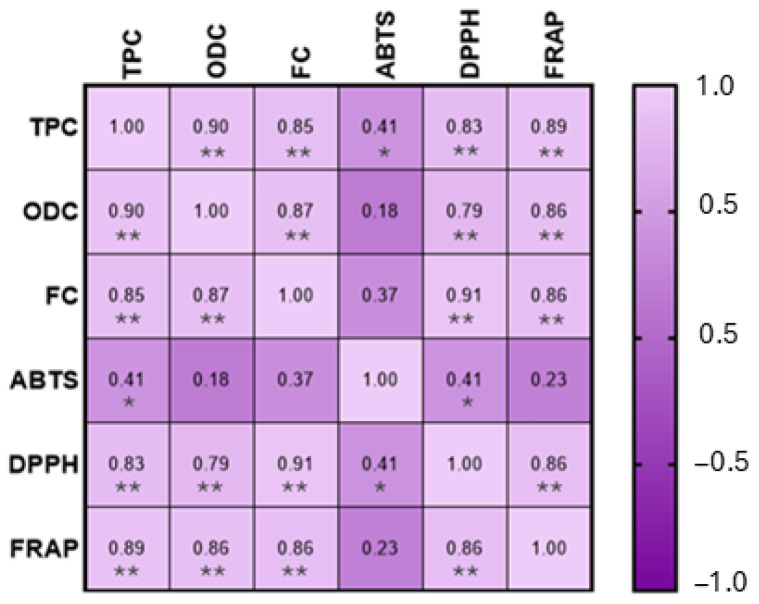
Pearson correlation between phenolic composition and antioxidant capacity. MS. Statistically significant correlations: * *p* < 0.05, ** *p* < 0.01.

A Pearson correlation was performed, in order to establish the correlation between the phenolic composition and the antioxidant capacity of the honey samples, as shown in Figure 3.

This analysis demonstrated a positive and significant correlation (*p* < 0.01), for the phenolic composition and the DPPH assay (total phenols: rDPPH = 0.83; *ortho*-diphenols: rDPPH = 0.79; flavonoids: rDPPH = 0.91) and FRAP assay (total phenols: rFRAP = 0.89; *ortho*-diphenols: rFRAP = 0.86; flavonoids: rFRAP = 0.86). Regarding the ABTS assay, a significant correlation (*p* < 0.05) was observed between ABTS and total phenols; however, no significant correlations were found between ABTS and *ortho*-diphenols and flavonoids. These results are corroborated by the data obtained for phenolic content (Table 1) and for the antioxidant capacity (Table 2), where the correlation between both is clear. This situation is also presented by Cheung et al. [55] and Cianciosi et al. [10]. The effectiveness of flavonoids has already been mentioned as the most important functional component of honey, and this parameter also provides a substantial contribution to honey’s total antioxidant activity and also its phenolic acids [56].

Interestingly, a negative correlation was observed between antioxidant activity measured by the ABTS assay and both the total phenolic content (TPC) and DPPH results. This apparent inconsistency can be explained by the differences in the chemical principles and reaction mechanisms of the assays used [57,58,59,60]. The ABTS assay is more responsive to hydrophilic antioxidants, whereas the DPPH assay preferentially reacts with lipophilic compounds. Moreover, different phenolic compounds exhibit varying affinities toward each radical type, which can lead to divergent assay outcomes. In complex matrices such as honey, interactions among antioxidant compounds may also influence the results, potentially causing synergistic or antagonistic effects that affect each assay differently. Therefore, the negative correlation does not necessarily indicate a lower antioxidant capacity but rather reflects the specific sensitivities of each analytical method. This highlights the importance of using multiple approaches to obtain a more comprehensive assessment of antioxidant activity [61,62].

**Figure 4:**
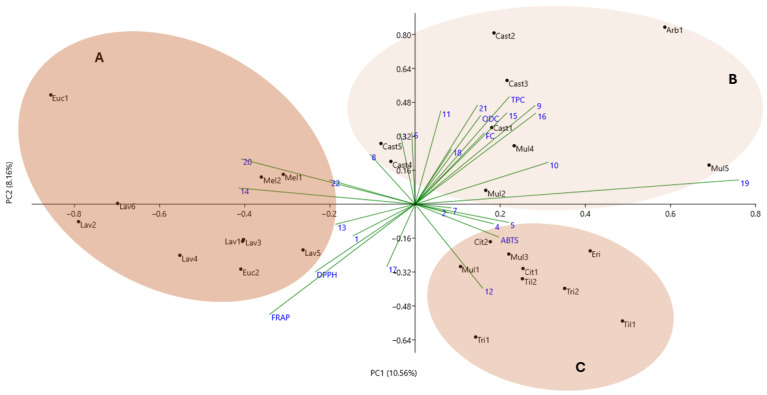
Canonical correspondence analysis (CCA) scatter plot applied to the phenolic compounds, antioxidant capacities, and molecular markers of the honey samples. Three different groups were identified based on botanical origin.

Canonical correspondence analysis (CCA) was carried out on the honey samples to analyze parameters related to phenolic composition, biological activities, and molecular markers and to infer possible relationships between honey samples from different geographical and botanical origins (Figure 4). The first two components of CCA accounted for 18.72% (PC1 = 10.56% and PC2 = 8.16%) of the loading scores for the seven parameters evaluated (total phenols, *ortho* diphenols, flavonoids, DPPH, ABTS, FRAP, and ISSR marker data). A total of three independent groups were obtained for this analysis.

Group A is composed by *Lavandula stoechas*, honeydew, and *Eucalyptus* spp. honey samples. The *Lavandula stoechas* samples are grouped, indicating genetic homogeneity within this species. On the other hand, the Euc1 sample is isolated and more distant, which could be related to the different genetic and chemical composition compared to other types of honey. The ABTS assay may be one of the parameters that contribute to this closeness.

Group B is composed of the chestnut samples, the *Arbutus unedo* honey sample, and the Mul2, Mul3, and Mul5 honey samples. This distribution is related to total phenols, *ortho*-diphenols, flavonoids, DPPH, and FRAP parameters. The samples of Cast1 and Cast3 are close to Mul4 probably related to a multifloral honey composition that has 77% of pollen from *Castanea sativa*.

The distribution of *Trifolium* spp., *Tilia* spp., *Erica umbellata*, *Citrus sinensis*, and Mul1 and Mul3 samples comprise group C, and their distribution is more related to the ISSR molecular marker data.

Honey samples with different botanical compositions also reflect the main botanical species, indicating that phenolic composition, biological activities, and molecular markers are effective in differentiating the botanical origins of honeys, making this analysis essential for differentiating and authenticating honeys based on their botanical origin.

## 3. Materials and Methods

### 3.1. Sampling

For this research, twenty-two monofloral honeys, five multifloral honeys, and two honeydew samples were used. Their geographical and botanical origins are detailed in Table 4 and Supplementary File S1. The honey samples were obtained from Apismaia (Póvoa de Varzim, Portugal), a company specializing in pollen analysis and physicochemical analyses, in 2023, and were stored at low temperature (0 to 25 °C). To determine the botanical origin of honey, the melissopalynology methodology of the International Honey Commission was used [63]. The samples were observed under a microscope (Olympus C33; Tokyo, Japan) and through an immersion lens (100×. At least 500 pollen grains from nectariferous plants were counted. To help identify some pollen grains, a comparison was performed using a laboratory pollen collection, the PalDat-Palynological Database (https://www.paldat.org/; accessed on 12 January 2025), and Valdéz et al. [64]. In general, a honey is considered monofloral if the relative frequency of the pollen type of that plant exceeds 45%. In this study, we had some overrepresented pollen types, *Castanea sativa* and *Eucalyptus globulus* (>70%), and underrepresented pollen types such as *Lavandula stoechas* (10–15%), *Citrus* spp. (>10%), and *Arbutus unedo* (>8%). In case of doubt about their botanical origin after pollen analysis, some samples underwent sensory and electrical conductivity analyses. These analyses were important to confirm honeydew and chestnut honeys. In this study, five multifloral honeys were used with a pollen analysis being performed (Table 5).

### 3.2. Phytochemical and Antioxidant Capacity Determination

#### 3.2.1. Sample Preparation

For sample preparation, 4 g of each honey sample was mixed with 50 mL of methanol/water (20:80, *v*/*v*) mixture. To determine the phenolic content and antioxidant capacity, the mixture was homogenized and extracted according to a previously described method by Santos et al. [65]. Subsequently, the samples were stored at 4 °C, with the remaining portions kept at −80 °C. In all tests performed, each sample was analyzed in triplicate (*n* = 3).

#### 3.2.2. Phenolic Content of Honey

For the quantification of the phenolic composition of honey, total phenols, *ortho*-diphenols, and flavonoids were determined using spectrophotometric methods. These methods were adapted to a microscale format using 96-well microplates (Frilabo, Milheirós, Portugal), and absorbance measurements were recorded using a Multiskan GOMicroplate Photometer (Thermo Fisher Scientific, Vantaa, Finland). Data were reported as the mean value of three replicates ± standard deviation.

The total phenol content in the honey samples was quantified using the Folin–Ciocalteau colorimetric method, following the methodology previously described by Gouvinhas et al. [66] with some adaptations. The standard curve was initially prepared using different concentrations of GA. A total of 20 µL of each standard solution and respective samples were added directly to each well, followed by 100 µL of the Folin–Ciocalteau reagent, pre-diluted with water (1:10 H_2_O). Afterward, 80 µL of a 7.5% sodium carbonate (Na_2_CO_3_) solution was added. The mixtures were incubated in an oven at 40–45 °C for 30 min, protected from light. The absorbance was measured at a wavelength of 750 nm using a microplate reader (Multiskan GOMicroplate Photometer, Thermo Fisher Scientific, Vantaa, Finland).

The *ortho*-diphenols content in the honey samples was determined using the colorimetric method previously described by Barros et al. [67], with slight adaptations. Firstly, 160 µL of each standard solution and respective samples were added directly to each well, followed by 40 µL of sodium molybdate (Na_2_MoO_4_) (5%). Then, the reaction was incubated at room temperature and protected from light for 15 min. The absorbance was measured at a wavelength of 375 nm.

For both assays, gallic acid was used as a standard. The results are expressed in mg of gallic acid (GA)/100 g of the sample.

To determine the flavonoid content in honey samples, the aluminum complex colorimetric method was employed according to Costa et al. [68]. Sodium nitrite (28 µL) was added to honey samples (24 µL) and incubated at room temperature for 5 min. Then, 10% aluminum chloride (28 µL) was added to the mixture. After 6 min, 1 M sodium hydroxide (120 µL) was added, and the absorbance of the mixture was measured at 510 nm after agitation for 30 s in a microplate reader. The flavonoid content was expressed as mg of catechin equivalents (CAT)/100 g of sample.

#### 3.2.3. Evaluation of the Antioxidant Capacity of Honey

For the evaluation of the antioxidant capacity of honey samples, ABTS, DPPH, and FRAP were determined using spectrophotometric methods, according to the methodology described by Barros et al. [67], with some adaptations.

In the ABTS assay, an ABTS working solution (188 µL) and sample dilutions (12 µL) were mixed and incubated for 30 min at room temperature. Absorbance was measured at 734 nm.

For the DPPH assay, 190 µL of DPPH solution was added to each well, followed by 10 µL of each standard solution or sample. The mixture was incubated at room temperature, protected from light, for 30 min, and the antioxidant capacity was then assessed by measuring the absorbance at 520 nm.

For the FRAP assay, 20 µL of each standard solution and samples were added to the wells, followed by 180 µL of working FRAP solution, composed of 10-volume acetate buffer (300 mM, pH = 3.6), 1-volume TPTZ (10 mM dissolved in hydrochloric acid), and 1-volume ferric chloride (20 mM in water). The mixture was shaken and incubated at 37 °C in the dark for 30 min. The absorbance was measured at 593 nm.

The results in these three methods were expressed as mmol Trolox equivalent per 100 g of sample (mmol Trolox/100 g), with data reported as the mean value of three replicates ± standard deviation.

### 3.3. Evaluation of Honey Sample’s Genetic Diversity

#### 3.3.1. DNA Extraction and Quantification

For the DNA extraction, 10 g of each honey sample was weighed and combined with 2 mL of water and 12 mL of 1× PBS (phosphate-buffered saline). The samples were then incubated at 65 °C for 10 min in a water bath, followed by centrifugation at 4500 rpm for 20 min. The supernatant was discarded, and the pellet from each sample was transferred to a 2 mL Eppendorf tube to be used for DNA extraction. The sample preparation was performed based on a protocol proposed by Soares et al. [69] with some modifications.

DNA extraction was performed using the CTAB-based method following the protocol described by Soares et al. [69], with some modifications—namely the incubation time during lysis (65 °C, 2 h) and the time to DNA precipitation being overnight at −20 °C. The yield and purity of the DNA extracted were assessed by spectrophotometry using the spectrophotometer Powerwave XS2 (BioTek Instruments, Inc., Winooski, VT, USA). The absorbance was read at 260 and 280 nm to estimate DNA content and purity using the nucleic acid quantification protocol. The A_260_/A_280_ ratio allowed the assessment of contaminations with proteins and/or RNA.

#### 3.3.2. ISSR-PCR

For ISSR analysis, a set of 25 ISSR primers (UBC#100/9) were initially screened, and 11 ISSR primers were selected (Table 4) according to their clear and reproducible band patterns and the high level of polymorphism. PCR amplification was performed in a final volume of 20 μL containing 3 μL of DNA template (10 ng/μL), 2 μL of Taq buffer with (NH_4_)_2_SO_4_ (10×), 1.6 μL of MgCl_2_ (25 mM), 2 μL of ISSR primer (5 μM), 0.5 μL of dNTPs (10 mM each), 0.4 μL of Taq polymerase (5 U/μL) (Thermo Scientific, Waltham, MA, USA), and 10.5 μL of H_2_O. The PCR reaction was programmed in a thermocycler (BioRad T100TM Thermal Cycler, Hercules, CA, USA), initiating with a denaturation step at 94 °C for 5 min followed by 45 cycles of 94 °C for 30 s, 52 °C for 45 s, 72 °C for 2 min, with a final extension at 72 °C for 10 min. The PCR products were analyzed on 1.5% agarose gel in 1× TBE buffer, running at 120 V for approximately 120 min. To determine the size of the PCR products, a 100 bp Plus DNA Ladder (Thermo Scientific, Waltham, MA, USA) was used in each agarose gel. DNA banding patterns generated by ISSR markers were obtained using the Molecular Image Gel-DocTM XR+ with Image LabTM Software v3.01 (BioRad, Hercules, CA, USA).

#### 3.3.3. Data Analysis

##### Statistical Analysis

Data were reported as the mean value of three replicates ± standard deviation (*n* = 3) and were analyzed in SPSS Statistics, version 29.0.0.0 (IBM SPSS Statistics Software, Chicago, IL, USA). Analysis of variance (ANOVA), followed by a post hoc Tukey test (*p*-value < 0.05), was carried out to detect differences between the content of total phenolics, *ortho*-diphenols, flavonoids, FRAP, ABTS, and DPPH. Correlation analysis (Pearson’s coefficient, *r*-value) was performed to understand the effect of the chemical composition on the bioactivity of the honey samples. In addition, canonical correspondence analysis (CCA) was carried out to highlight the chemical composition, biological activities, and molecular markers and to infer possible relationships between honey samples from different geographical and botanical origins. Canonical correspondence analysis was performed in Past version 3.19 statistical software [70], using values normalized into percentages considering the maximum value obtained for each assay/test. Correlation was performed in GraphPad Prism, version 10.2.3 (GraphPad Software, San Diego, CA, USA).

ISSR is a dominant marker, it being assumed that each band was considered to represent a single bi-allelic locus, and each amplified band/marker was scored as present (1) or absent (0). Each primer–sample combination was repeated at least twice, and only reproducible bands were used for analysis. The ISSRs were analyzed by determining the number of polymorphic bands (NPB), unique bands (NUB), and the total number of bands (TNB), as well as calculating the percentage of polymorphism (P%). Following the parameters presented in Carvalho et al. [24], the resolution power (Rp) was calculated with the formula Rp = ∑ Ib (Ib = 1 − [2 × (0.5 − pi), with Ib being the band information and pi the proportion of individuals containing the band. The polymorphic information content (PIC) was determined with the formula PICi = 2 fi (1 − fi), with Fi being the frequency of the amplified band. The product of polymorphism percentage and PIC was designated as the marker index (MI).

## 4. Conclusions

Honey is a high-value food product that has emerged as a natural food source of phenolic compounds with biological properties, which are typically associated with its chemical composition. Therefore, it is essential to consider the geographical and botanical origins of honey, as these factors influence its quality for consumers, as well as its physical, chemical, organoleptic, and bioactive characteristics. These attributes serve as valuable markers for determining honey’s quality and authenticity.

This study focused on the potential of Portuguese monofloral honeys from different botanical and geographical origins by evaluating their chemical compositions, especially regarding secondary metabolites, and assessing the relationship between their molecular and phytochemical characteristics. The results showed that, in general, geographical origin does not appear to significantly influence the phenolic composition and antioxidant capacity of the analyzed honeys. In contrast, botanical origin had a more pronounced impact on these parameters.

The findings revealed a higher phenolic content in certain types of honey, such as honeydew, multifloral, and specific monofloral varieties, including *Castanea sativa*, *Arbutus unedo*, and *Erica umbellata*. These honeys, especially those from the north and center of Portugal, presented higher concentrations of phenolic compounds and greater antioxidant capacity. The analysis also confirmed a positive and significant correlation between phenolic content and antioxidant capacity. Additionally, this study evaluated the effectiveness of ISSR markers in differentiating the botanical origin of honey. The ISSR molecular marker allows the detection of different degrees of genetic diversity among the honey samples, demonstrating its moderate value in studies of genetic diversity and botanical origin discrimination within this food matrix. In general, the honey samples were grouped based on their botanical origin. In the future, it would be valuable to use additional molecular markers, such as chloroplast SSR markers, to further confirm the botanical origin and enhance honey traceability. These findings would be useful to establish new approaches for honey adulteration detection, authenticity, and quality control.

The parameters studied proved to be effective in distinguishing the botanical origin of honey. However, clear differentiation based on geographical origin was not realized. In conclusion, this multidisciplinary study highlights that honey contains essential bioactive compounds, such as phenolic compounds and antioxidants, which contribute significantly to the value of this food product.

## Figures and Tables

**Table 1:** Phenolic content indexes in the 29 Portuguese honey samples. Total phenolics (TPC) and *ortho*-diphenols (ODC) expressed as mg of gallic acid equivalent (GAE) in 100 g of honey (g); flavonoids (FC) expressed as mg of catechin equivalent (CE) in 100 g of honey (g). The values are presented as the mean ± standard deviation (*n* = 3). Significant differences were evaluated by one-way ANOVA, followed by Tukey’s tests for parameters. Means followed by different letters within the same column are significantly different (*p* < 0.05).

Botanical Origin	Sample	TPC mg GAE/100 g	ODC mg GAE/100 g	FC mg CE/100 g
***Lavandula*** ***stoechas***	Lav1	42.15 ± 0.87 ^m^	53.30 ± 1.14 ^kl^	3.87 ± 0.48 ^g–j^
	Lav2	40.97 ± 0.55 ^mn^	58.35 ± 0.39 ^jk^	3.52 ± 0.19 ^h–j^
	Lav3	28.88 ± 0.40 ^qr^	41.59 ± 0.35 ^m–o^	3.09 ± 0.12 ^i–k^
	Lav4	48.14 ± 1.13 ^l^	41.51 ± 0.17 ^m–o^	2.70 ± 0.087 ^j–l^
	Lav5	24.61 ± 0.60 ^rs^	35.75 ± 0.51 ^op^	3.05 ± 0.34 ^i–k^
	Lav6	34.34 ± 0.21 ^op^	40.78 ± 0.94 ^m–o^	3.73 ± 0.34 ^g–j^
***Castanea*** ***sativa***	Cast1	53.75 ± 1.35 ^k^	61.18 ± 0.28 ^i–k^	4.04 ± 0.23 ^g–j^
	Cast2	62.02 ± 2.31 ^ij^	65.25 ± 0.51 ^g–j^	3.70 ± 0.31 ^h–j^
	Cast3	71.01 ± 0.64 ^h^	88.09 ± 0.90 ^d^	4.19 ± 0.32 ^f–j^
	Cast4	77.36 ± 3.44 ^fg^	64.38 ± 2.63 ^h–j^	4.48 ± 0.31 ^f–i^
	Cast5	86.68 ± 0.81 ^de^	72.95 ± 1.29 ^fg^	4.75 ± 0.78 ^e–h^
***Tilia* spp.**	Til1	35.06 ± 2.11 ^op^	40.94 ± 2.98 ^m-o^	3.60 ± 0.21 ^h–j^
	Til2	31.48 ± 0.02 ^pq^	35.78 ± 0.54 ^op^	4.30 ± 0.20 ^f–i^
***Citrus*** ***sinensis***	Cit1	20.13 ± 0.37 ^s^	19.73 ± 0.59 ^q^	1.40 ± 0.18 ^l^
	Cit2	27.05 ± 0.15 ^qr^	30.56 ± 0.75 ^p^	1.92 ± 0.76 ^kl^
***Eucalyptus*** **spp.**	Euc1	36.61 ± 0.36 ^no^	45.83 ± 0.40 ^l–n^	3.71 ± 1.01 ^h–j^
	Euc2	49.59 ± 0.32 ^kl^	39.70 ± 0.46 ^no^	4.42 ± 0.43 ^f–i^
***Trifolium*** **spp.**	Tri1	34.41 ± 0.43 ^op^	48.09 ± 0.66 ^lm^	3.55 ± 0.10 ^h–j^
	Tri2	45.32 ± 1.35 ^lm^	48.34 ± 1.40 ^lm^	3.45 ± 0.10 ^h–j^
***Erica umbellata***	Eri1	95.72 ± 1.19 ^c^	120.22 ± 6.39 ^b^	8.68 ± 0.37 ^d^
**Honeydew**	Mel1	105.10 ± 0.96 ^b^	98.14 ± 0.88 ^c^	14.49 ± 0.24 ^b^
	Mel2	126.00 ± 0.29 ^a^	188.73 ± 1.07 ^a^	19.43 ± 0.42 ^a^
***Arbutus unedo***	Arb	91.42 ± 0.48 ^cd^	70.34 ± 0.83 ^gh^	10.76 ± 0.67 ^c^
***Metrosíderos excelsa***	Met	27.48 ± 1.38 ^qr^	33.92 ± 0.08 ^op^	1.66 ± 0.15 ^kl^
**Multifloral**	Mul1	59.70 ± 0.24 ^j^	66.89 ± 1.10 ^g–i^	5.70 ± 0.31 ^ef^
	Mul2	74.02 ± 0.91 ^gh^	78.91 ± 0.42 ^ef^	10.45 ± 1.17 ^c^
	Mul3	65.14 ± 2.34 ^i^	80.27 ± 10.87 ^d–f^	5.24 ± 0.28 ^e–g^
	Mul4	82.89 ± 0.13 ^e^	82.56 ± 1.36 ^de^	6.16 ± 0.11 ^e^
	Mul5	82.10 ± 5.23 ^ef^	97.48 ± 2.02 ^c^	10.05 ± 0.88 ^cd^

**Table 2:** In vitro antioxidant activities (ABTS, DPPH, and FRAP) in the 29 Portuguese honey samples and the scavenging capacity (%) for the DPPH and ABTS methods. Activities expressed as mmol Trolox per 100 g of honey. The values are presented as the mean ± standard deviation (*n* = 3). Significant differences evaluated by one-way or two-way ANOVA, followed by Tukey tests. Letters indicate significant differences between honey samples (*p* < 0.05).

Botanical Origin	Sample	ABTS	ABTS (%)	DPPH	DPPH (%)	FRAP
		**mmol Trolox/100 g**		**mmol Trolox/100 g**		**mmol Trolox/100 g**
***Lavandula stoechas***	Lav1	0.38 ± 0.02 ^d^	14.44	0.058 ± 0.00 ^o^	7.15	0.11 ± 0.001 ^jk^
	Lav2	0.30 ± 0.019 ^e^	11.61	0.11 ± 0.011 ^kl^	10.30	0.15 ± 0.006 ^j^
	Lav3	0.15 ± 0.004 ^h–k^	28.95	0.036 ± 0.002 ^p^	5.87	0.064 ± 0.002 ^l^
	Lav4	0.16 ± 0.004 ^g–j^	31.24	0.041 ± 0.003 ^p^	6.13	0.063 ± 0.001 ^l^
	Lav5	0.10 ± 0.003 ^lm^	19.23	0.022 ± 0.002 ^q^	5.07	0.046 ± 0.002 ^l^
	Lav6	0.15 ± 0.004 ^h–k^	28.41	0.14 ± 0.002 ^h^	13.09	0.11 ± 0 ^jk^
***Castanea sativa***	Cast1	0.11 ± 0.005 ^lm^	17.88	0.081 ± 0.003 ^n^	7.38	0.32 ± 0.006 ^gh^
	Cast2	0.14 ± 0.013 ^j–l^	23.09	0.093 ± 0.00 ^mn^	8.17	0.34 ± 0.018 ^fg^
	Cast3	0.15 ± 0.008 ^i–k^	24.45	0.14 ± 0.004 ^gh^	11.81	0.47 ± 0.009 ^de^
	Cast4	0.16 ± 0.003 ^g–j^	27.20	0.13 ± 0.005 ^hi^	11.42	0.38 ± 0.053 ^f^
	Cast5	0.53 ± 0.021 ^c^	18.91	0.18 ± 0.002 ^f^	17.03	0.22 ± 0.005 ^i^
***Tilia* spp.**	Til1	0.095 ± 0.007 ^mn^	15.09	0.09 ± 0.002 ^n^	7.87	0.20 ± 0.018 ^i^
	Til2	0.24 ± 0.001 ^f^	47.77	0.14 ± 0.004 ^gh^	13.47	0.15 ± 0.001 ^j^
***Citrus sinensis***	Cit1	0.059 ± 0.001 ^no^	8.79	0.028 ± 0.002 ^pq^	2.22	0.046 ± 0.003 ^l^
	Cit2	0.12 ± 0.01 ^k–m^	20.93	0.12 ± 0.005 ^i–k^	11.56	0.081 ± 0.001 ^kl^
***Eucalyptus* spp.**	Euc1	0.24 ± 0.003 ^f^	49.11	0.062 ± 0.002 ^o^	5.63	0.12 ± 0 ^jk^
	Euc2	0.80 ± 0.028 ^a^	31.13	0.15 ± 0.005 ^g^	14.56	0.15 ± 0.003 ^j^
***Trifolium* spp.**	Tri1	0.19 ± 0.01 ^gh^	36.62	0.12 ± 0.007 ^j–l^	10.59	0.13 ± 0.008 ^j^
	Tri2	0.19 ± 0.01 ^g^	37.39	0.061 ± 0.002 ^o^	7.32	0.083 ± 0.004 ^kl^
***Erica umbellata***	Eri	0.24 ± 0.012 ^f^	6.27	0.24 ± 0.003 ^d^	23.21	0.36 ± 0.013 ^fg^
**Honeydew**	Mel1	0.69 ± 0.024 ^b^	26.28	0.39 ± 0.005 ^b^	38.23	0.66 ± 0.002 ^b^
	Mel2	0.19 ± 0.012 ^g–i^	3.91	0.47 ± 0.004 ^a^	45.83	0.77 ± 0.025 ^a^
***Arbutus unedo***	Arb	0.40 ± 0.01 ^d^	13.39	0.37 ± 0.006 ^c^	35.85	0.50 ± 0.005 ^cd^
***Metrosíderos excelsa***	Met	0.038 ± 0.004 ^o^	5.31	0.11 ± 0.002 ^lm^	9.06	0.15 ± 0.002 ^j^
**Multifloral**	Mul1	0.33 ± 0.018 ^e^	10.58	0.11 ± 0.002 ^lm^	9.98	0.23 ± 0.002 ^i^
	Mul2	0.40 ± 0.011 ^d^	13.52	0.20 ± 0.001 ^e^	19.72	0.36 ± 0.003 ^f^
	Mul3	0.18 ± 0.002 ^g–j^	31.70	0.18 ± 0.002 ^f^	15.68	0.44 ± 0.03 ^e^
	Mul4	0.38 ± 0.018 ^d^	12.71	0.13 ± 0.001 ^h–j^	12.30	0.28 ± 0.004 ^h^
	Mul5	0.18 ± 0.009 ^g-i^	29.89	0.20 ± 0.01 ^e^	16.35	0.51 ± 0.006 ^c^

**Table 3:** Inter simple sequence repeat (ISSR) polymorphism parameters obtained from the 28 Portuguese honey samples.

Primer	Sequence	TNB	NPB	NUB	%P	Size Range (bp)	Rp	PIC	MI
UBC-810	(GA)_8_T	10	10	1	100	800–200	6.93	0.34	34.31
UBC-811	(GA)_8_C	12	12	2	100	1000–350	6.64	0.35	34.80
UBC-824	(TC)_8_G	9	9	1	100	1600–350	5.43	0.36	36.05
UBC-826	(AC)_8_T	9	9	0	100	1800–400	7.64	0.42	41.58
UBC-836	(AG)_8_YA	14	14	0	100	1000–300	7.57	0.32	32.29
UBC-840	(GA)_8_YT	12	9	0	75	1500–300	6.86	0.29	22.00
UBC-856	(AC)_8_YA	13	13	0	100	1700–300	8.07	0.37	36.79
UBC-857	(AC)_8_YG	10	10	2	100	2000–300	4.29	0.27	26.73
UBC-873	(GACA)_4_	9	9	1	100	1600–400	6.86	0.31	31.29
UBC-888	BDB(CA)_7_	11	11	0	100	2000–390	9.71	0.43	42.72
UBC-891	HVH (TG)_7_	9	9	0	100	1800–300	6.29	0.39	39.00
	Average	10.73	10.45	0.64	97.73		6.94	0.35	34.33

TNB = total number of bands; NPB = number of polymorphic bands; NUB = number of unique bands; P% = polymorphism percentage; Rp = resolution power; PIC = polymorphism information content; MI = marker index.

**Table 4:** List of honey samples used in this study and the details of botanical source and geographical origin.

Botanical Origin	Codification	Geographical Origin	District
***Lavandula stoechas***	Lav1	Sines	Setúbal
	Lav2	Chamusca	Santarém
	Lav3	Messines	Faro
	Lav4	Mirandela	Bragança
	Lav5	Almodôvar	Beja
	Lav6	Tavira	Faro
***Castanea sativa***	Cast1	Viseu	Viseu
	Cast2	Satão	Viseu
	Cast3	Viseu	Viseu
	Cast4	Vila Pouca Aguiar	Vila Real
	Cast5	Cinfães	Viseu
***Tilia* spp.**	Til1	Tondela	Viseu
	Til2	Oliveira do Hospital	Coimbra
***Citrus sinensis***	Cit1	Silves	Faro
	Cit2	Tavira	Faro
***Eucalyptus* spp.**	Euc1	Espinho	Aveiro
	Euc2	Arouca	Aveiro
***Trifolium* spp.**	Tri1	Alcains	Castelo Branco
	Tri2	Castelo Branco	Castelo Branco
***Erica umbellata***	Eri1	Seia	Guarda
**Honeydew**	Mel1	Almeida	Guarda
	Mel2	Vinhais	Bragança
***Metrosíderos excelsa***	Met1	Aveiro	Aveiro
***Arbutus unedo***	Arb1	Monchique	Faro
**Multifloral**	Mul1	Freamunde	Porto
	Mul2	Celorico da Beira	Guarda
	Mul3	Chaves	Vila Real
	Mul4	Gois	Coimbra
	Mul5	Mirandela	Bragança

**Table 5:** Pollen analysis of the five multifloral honey samples under study.

Sample	Main Pollen Types (%)
**Mul1**	*Castanea sativa* (65%); *Rubus* spp. (15%); *Eucalyptus globulus* (10%)
**Mul2**	*Echium plantagineum* (46%); *Trifolium* spp. (11%)
**Mul3**	*Castanea sativa* (53%); *Echium plantagineum* (18%); *Rubus* spp. (14%)
**Mul4**	*Castanea sativa* (77%); *Erica* spp. (9%); *Rubus* spp. (6%)
**Mul5**	*Castanea sativa* (59%); *Rubus* spp. (38%)

## Data Availability

The original contributions presented in this study are included in the article/Supplementary Material. Further inquiries can be directed to the corresponding author(s).

## Supplementary Materials

The following supporting information can be downloaded at: https://www.mdpi.com/article/10.3390/molecules30081808/s1, File S1: Geographical localization of the honey samples used in the study.
