# Supplementary material for: Portuguese Monofloral Honeys: Molecular Insights and Biochemical Characterization
Source: Molecules. 2025 Apr 17;30(8):1808. doi: 10.3390/molecules30081808 (PMC12029150; doi:10.3390/molecules30081808)

Supplementary file

Supplementary file S1 – Geographical localization of the honey samples used in the study.

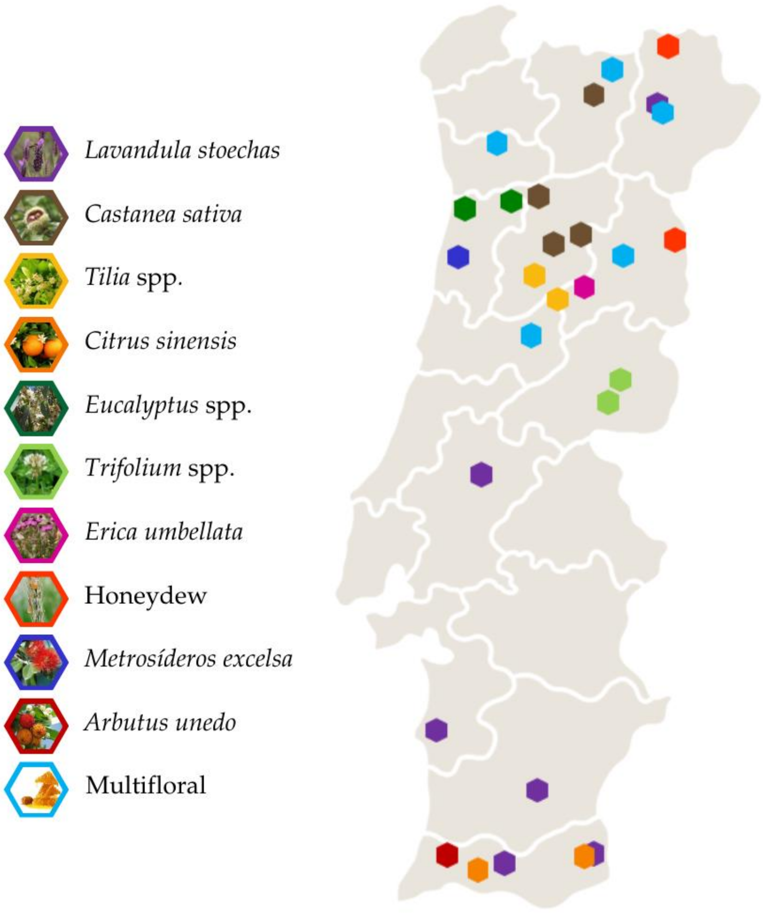

Supplement: Supplementary file 1 [file molecules-30-01808-s001.zip › molecules-3559875-supplementary.pdf]
